# Improving Dorsal Stream Function in Dyslexics by Training Figure/Ground Motion Discrimination Improves Attention, Reading Fluency, and Working Memory

**DOI:** 10.3389/fnhum.2016.00397

**Published:** 2016-08-08

**Authors:** Teri Lawton

**Affiliations:** ^1^Perception Dynamics InstituteDel Mar, CA, USA; ^2^Department of Computer Science and Engineering, University of California, San DiegoLa Jolla, CA, USA

**Keywords:** attention networks, reading remediation, cortical plasticity, perceptual learning, improving dorsal stream function, neural timing, dyslexia development, figure/ground motion discrimination

## Abstract

There is an ongoing debate about whether the cause of dyslexia is based on linguistic, auditory, or visual timing deficits. To investigate this issue three interventions were compared in 58 dyslexics in second grade (7 years on average), two targeting the temporal dynamics (timing) of either the auditory or visual pathways with a third reading intervention (control group) targeting linguistic word building. Visual pathway training in dyslexics to improve direction-discrimination of moving test patterns relative to a stationary background (figure/ground discrimination) significantly improved attention, reading fluency, both speed and comprehension, phonological processing, and both auditory and visual working memory relative to controls, whereas auditory training to improve phonological processing did not improve these academic skills significantly more than found for controls. This study supports the hypothesis that faulty timing in synchronizing the activity of magnocellular with parvocellular visual pathways is a fundamental cause of dyslexia, and argues against the assumption that reading deficiencies in dyslexia are caused by phonological deficits. This study demonstrates that visual movement direction-discrimination can be used to not only detect dyslexia early, but also for its successful treatment, so that reading problems do not prevent children from readily learning.

## Introduction

Dyslexia is a multifaceted reading disability that encompasses both pronunciation-based and visual processing-based reading issues (Stein, 2001) that is characterized by severe reading and spelling problems (Vellutino et al., 2004). Reading difficulties, including people having dyslexia and attention deficits, are prevalent in the United States (US) where 65% of fourth graders and 62% of 12th graders are not proficient in reading (National Center for Educational Statistics, 2013). Previous studies have shown that reading difficulties in many children may *indeed* be prevented through early intervention (Schatschneider et al., 2004). Identification of the cognitive skills that predict subsequent reading ability can help identify children at risk for reading problems, and following appropriate training reduce the severity of their symptoms (Kevan and Pammer, 2009). Since motion detection deficits in pre-reading children predict who will develop reading problems (Boets et al., 2011), it is likely that a task to improve motion discrimination, and thereby timing, in either the auditory or visual domain will remediate reading problems, a key question addressed by this study.

Slow reading speeds are a hallmark of dyslexia (Lyon et al., 2003; Nicholson and Fawcett, 2007). Children with dyslexia are reported to have some combination of spatial (Lovegrove et al., 1980; Cornelissen et al., 1995; Stein and Walsh, 1997; Lawton, 2000, 2007, 2008, 2011; Talcott et al., 2000; Hansen et al., 2001; Stein, 2001) and/or temporal (Stanley and Hall, 1973; Bradley and Bryant, 1983; Tallal et al., 1993; Temple et al., 2003) sequencing deficits. These spatial and temporal sequencing deficits cause the letters in the words and the words on the page to appear distorted, displaced, or crowded together (Atkinson, 1991), often resulting in eyestrain and headaches (Wilkins, 1995). These spatial and temporal sequencing deficits, found when images are rapidly presented or moving, have been hypothesized to result from neural timing deficits associated with sluggish magnocellular neurons (Livingstone et al., 1991; Stein and Walsh, 1997; Vidyasagar, 1999, 2001, 2012; Lawton, 2000, 2007, 2008, 2011; Stein, 2001; Vidyasagar and Pammer, 2010; Boets et al., 2011), causing deficits in integration of information between magnocellular (“where”) and parvocellular (“what”) neurons. A normally functioning magnocellular pathway is sensitive to *low-contrast* achromatic patterns (Kaplan and Shapley, 1986; Sclar et al., 1990). All dyslexics exhibit high contrast thresholds for discriminating the direction of moving patterns against stationary background patterns (Lawton, 2000, 2007, 2011; Ridder et al., 2001).

### Visual Timing (Magnocellular) Deficits in Dyslexics

Receiving predominantly magnocellular input (Livingstone and Hubel, 1988; Maunsell et al., 1990; Merigan and Maunsell, 1993), the dorsal stream, specialized for processing the movement and location of objects in space (Ungerleider and Mishkin, 1982; Livingstone and Hubel, 1988; Felleman and Van Essen, 1991), projects from the primary visual cortex (V1), through visual area medial temporal cortex (MT), and on to the posterior parietal cortex (PPC), a selective spatial attention area (Posner et al., 1984). This is in contrast to the ventral stream which receives both magnocellular and parvocellular inputs as it projects from V1 through area V4 and on to the infero-temporal (IT) cortex, an area specialized in extracting details relating to an object’s shape and color (Ungerleider and Mishkin, 1982; Livingstone and Hubel, 1988; Felleman and Van Essen, 1991).

Dyslexics have magnocellular responses that were found to be 20–40 ms slower than typically developing observers (Livingstone et al., 1991), being 2–4 fold slower than the normal magnocellular lead time of 10 ms (Dreher et al., 1976). Some investigators hypothesize that in dyslexics a lack of synchronization in timing between magnocellular and parvocellular activations may prevent effective sequential processing, pattern analysis, and figure/ground discrimination, and hence impede development of efficient reading and attention skills (Stein and Walsh, 1997; Vidyasagar, 1999, 2001, 2012; Lawton, 2000, 2007, 2008, 2011, 2015; Stein, 2001). It is further possible that the dyslexic reader’s deficit in attentional focus (Vidyasagar, 1999, 2001; Facoetti et al., 2000, 2010; Solan et al., 2001) is another consequence of sluggish magnocellular neurons, preventing the linked parvocellular neurons from isolating and sequentially processing the relevant information needed for reading (Vidyasagar, 1999, 2001; Vidyasagar and Pammer, 2010), and not from the information overload as proposed previously (Stuart et al., 2001).

Visual timing deficits resulting from sluggish magnocellular (motion-sensitive) neurons in the dorsal stream are likely to be highly involved in the dyslexic’s reading deficits (Stein and Walsh, 1997; Vidyasagar, 1999, 2001; Lawton, 2000, 2007; Stein, 2001; Gori et al., 2014). Convergent evidence suggests that many dyslexic readers demonstrate impairments in tasks that require dorsal stream involvement. Dyslexics have been found to have deficits in motion perception at: (1) the retinal level (Tyler, 1974) when measured using the frequency doubling illusion (Pammer and Wheatley, 2001; Buchholz and McKone, 2004; Kevan and Pammer, 2009; Gori et al., 2014); (2) V1 measured using Visual Evoked Potentials (VEPs; Livingstone et al., 1991; Shelley-Tremblay et al., 2011); (3) V1 and MT using both fMRI brain imaging (Eden et al., 1996; Demb et al., 1998) and psychophysical tasks of movement discrimination relative to a stationary background (Lawton, 2000, 2007, 2011); (4) MT using motion coherence for direction discrimination (Cornelissen et al., 1995; Talcott et al., 2000; Hansen et al., 2001; Boets et al., 2011); (5) the lateral intraparietal cortex (LIP) and Frontal Eye Fields (FEF), anterior cortical areas activated by saccades, based on saccade and antisaccade training tasks (Fischer, 2012); and (6) parietal structures, prefrontal language systems, cerebellum, and basal ganglia (Nicholson and Fawcett, 2007). These results are consistent with the suggestion of a relationship between dorsal stream processing and reading ability, such that poor dorsal processing relates to slower timing and poor reading skills. This study demonstrates that when a figure/ground motion discrimination paradigm is used, then poor reading skills are not only associated with poor visual dorsal stream functioning, but also can be remediated rapidly by training designed to improve dorsal stream function.

The degree to which dorsal stream deficits play a *causal* role in reading failure has yet to be established (Boden and Giaschi, 2007; Kevan and Pammer, 2009). Previous results indicate that there is a relationship between dorsal stream sensitivity and reading skill found in both pre-kindergarten children before reading is learned (Kevan and Pammer, 2009) and after the emergence of reading in children (Boets et al., 2011) and adults. Intervention studies targeting dorsal stream function need to be carried out in order to establish a direct causal link from dorsal stream functioning to reading skill (Kevan and Pammer, 2009). It is possible that since visual movement-discrimination training, designed to improve dorsal stream function, caused the reading speeds of dyslexic children to increase up to 10 times faster (Lawton, 2011), training dorsal stream function may be essential for developing not only reading fluency, but also the attention networks. Therefore, this study will not only measure reading fluency, but also measure attention and both visual and auditory working memory, for the first time, using standardized tests to demonstrate the range of cortical areas affected by training aimed at improving function in the V1-MT dorsal stream areas.

**Figure 1:**
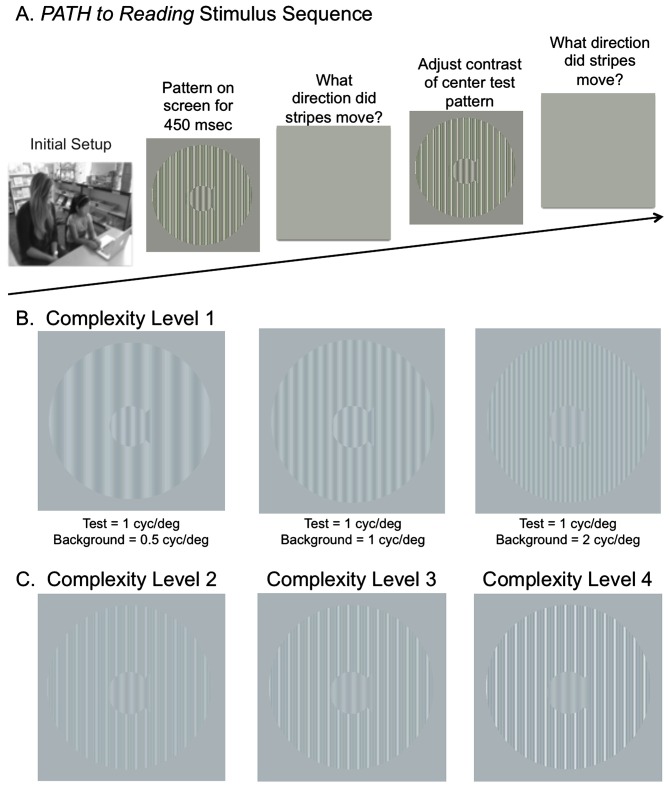
**(A)** Schematic of Stimulus Presentation for *PATH* *to Reading* intervention. Pattern flashes on screen (shown above) while center stripes move left or right. Screen goes blank, waits for left or right arrow key to be pushed. If incorrect, short tone sounds. Pattern with same or different contrast flashes on screen while center stripes move left or right. Screen goes blank, waits for left or right arrow key to be pushed. This sequence of patterns is presented continuously until the contrast threshold for this pattern is measured. Then the next pattern combination is presented to measure next contrast threshold … until all 20 *PATH* *to Reading* patterns were presented, and the program says “Thank You” and quits. **(B)** Sample patterns at Complexity Level 1 for a background one octave lower in spatial frequency (0.5 cyc/deg) than the test frequency, equal in spatial frequency to the test frequency (1 cyc/deg), and one octave higher in spatial frequency (2 cyc/deg) than the test frequency for a 1 cyc/deg “fish shaped” test pattern. **(C)** Sample patterns at Complexity Levels 2, 3, and 4 for the center pattern in **(B)**. These patterns have multifrequency background patterns (1 cyc/deg + 2 cyc/deg + 3 cyc/deg) for a 1 cyc/deg test pattern on a 5% (Complexity Level 2), 10% (Complexity Level 3), and 20% (Complexity Level 4) contrast background. These same four complexity levels are repeated at subsequently faster speeds for each set of four complexity levels, increasing from 6.7 Hz (complexity levels 1–4) to 8 Hz (complexity levels 5–8) to 10 Hz (complexity levels 9–12) to 13.3 Hz (complexity levels 13–16), as listed in Table 2.

The novel question addressed by this study is whether improving neural timing in the dorsal stream (by improving magnocellular function) improves reading fluency more when training is in the auditory domain to improve auditory timing (language-based), or is in the visual domain using a visual motion direction-discrimination task (improving visual timing), when compared to a traditional reading intervention, using linguistic word building that does not specifically target neural timing. The intervention we used to improve auditory timing lengthens the individual phonemes so that phonological processing improves, the length of the phonemes decreasing as the training progresses. Motion direction-discrimination training, on the other hand, measures the contrast needed for figure/ground discrimination of sinewave gratings moving left or right relative to a stationary background. These backgrounds increase task complexity by increasing the number of background spatial frequencies, background contrast, thereby activating more parvocellular neurons, with left-right movement increasing in speed as the training progresses. The motion direction-discrimination training patterns, vertical sinewave gratings (Figure 1), are designed to differentially activate motion-sensitive (magnocellular) neurons in the V1-MT network (Allman et al., 1985; Felleman and Van Essen, 1991; De Valois et al., 2000) relative to pattern-sensitive (parvocellular) neurons, thereby being an effective training stimulus to improve magno-parvo integration deficits at both early and higher levels of motion processing. Unlike the motion direction-discrimination training paradigm used in this study, direction-discrimination using motion coherence of random dots, differentially activates motion-sensitive neurons only in MT and at higher processing levels (Zohary et al., 1994; Braddick et al., 2001). Deficits in detecting motion coherence are rarely found in all individuals in a dyslexic sample (e.g., Talcott et al., 2013). Moreover, improvements using motion coherence direction-discrimination (Solan et al., 2004) have not been shown to be as effective a training paradigm to improve reading speed as found previously using direction-discrimination of dim vertical bars moving relative to a stationary textured background (Lawton, 2000, 2011).

This study explored the hypothesis that if sluggish magnocellular neurons underlie dyslexia, then training to improve the sensitivity and timing of magnocellular relative to parvocellular processing should improve reading fluency and attention. This study investigated whether improving dorsal stream function is more effective in remediating reading, attention, and memory problems when the intervention training improves timing in the auditory or visual domain, compared to linguistic methods for improving phonological processing. Since both the auditory timing and linguistic interventions required responses chosen from a larger number of possible responses, it is likely they require more frequent use of selective attention than the visual direction-discrimination task, providing good comparison interventions to determine whether visual training in the dorsal stream is the most effective type of training to improve attention.

## Materials and Methods

Only students who were diagnosed as being dyslexic by the Decoding Encoding Screener for Dyslexia (DESD), based on single word decoding (word identification) and encoding (spelling and writing phonetically), participated in this study. The DESD, standardized by Western Psychological Services in Los Angeles, CA, USA was clinically validated using the Woodcock-Johnson standardized reading tests (Guerin et al., 1993), as well as the Gray Oral Reading Test (GORT) and spelling subtest of the Wide Range Achievement Test (WRAT; Handford and Borsting, 2015). In this study, the severity of dyslexia, most having borderline or mild dyslexia: (scored as 1: above normal, 2: normal, 3: borderline dyslexia, 4: mild dyslexia, 5: moderately severe dyslexia, and 6: markedly below normal) was determined by combining each student’s dyseidetic score from 1 to 6 (spelling problems) and dysphonetic score from 1 to 6 (pronunciation problems) on the DESD. Matched samples were created by ordering students by their severity of dyslexia using this combined score, and then randomly assigning this ordered list into one of the three groups, either the control group or one of the two treatment groups, or in year-round schools into one of two groups, control and visual direction-discrimination training.

Dyslexic second graders (7 years old) were trained on three different reading interventions to improve: (1) auditory timing; (2) visual timing; or (3) linguistic word building. Second graders were studied since they are in the middle of the developmental period to learn direction discrimination (Lawton, 2000, 2007, 2008; Wolf et al., 2000), maximizing the ease of learning this task. This study was conducted in six elementary schools in San Diego Unified School District (SDUSD), where 50% of the students were reading below proficiency, as revealed by the California Standardized Tests available on each school’s website. The students spoke English fluently. This study was carried out in accordance with the recommendations of both the University of California San Diego (UCSD) IRB and The Research and Reporting Department at SDUSD with written informed consent from all subjects. All subjects gave written informed consent in accordance with the Declaration of Helsinki.

This study examined 58 children in second grade, 7.4 ± 0.4 years of age, 49% girls and 51% boys. The number of participants in Traditional Schools (TS) was seven control, six visual timing and six auditory timing students; and in year-round schools was 19 control and 20 visual timing students. Even though participants were randomly assigned from the ordered list of DESD scores into either two (year-round schedules) or three groups (TS schedules), the groups were balanced on age and baseline reading rate, attention and working memory, as shown in Table 1. The ethnic distribution for students in: (1) TS was 48.6% Caucasian, 25.7% Hispanic, 8.6% African American, and 17.1% Asian; and (2) Year-Round schools was 33.3% Caucasian, 28.2% Hispanic, 10.3% African American, and 28.2% Asian. These ethnicities were distributed equally among groups.

**Table 1:** **Subject baseline age and standardized scores for each group and significance levels across groups**.

Baseline values	Control	PATH	FFW	*F* value	*p* value
Age	7.4 ± 0.5	7.4 ± 0.4	7.3 ± 0.2	0.39	0.82
Reading rate (words/min)	145 ± 48	136 ± 50	110 ± 30	1.73	0.15
Reading comprehension	7.2 ± 2.1	6.3 ± 2.8	5.8 ± 2.0	0.70	0.59
Blending words	8.9 ± 1.3	9.2 ± 2.7	8.8 ± 2.0	0.18	0.95
Attention (CAS)	81.7 ± 8	79.9 ± 9	77.7 ± 8	1.40	0.24
Sequential visual memory	95 ± 10	97.7 ± 13.2	96.3 ± 7.5	0.40	0.81
Sequential auditory memory	92.8 ± 9.6	92.8 ± 9.6	89.8 ± 9	0.51	0.73
NonSeq auditory memory	92.8 ± 11	91.8 ± 8	87.2 ± 8	1.10	0.36
Delayed recall	91 ± 15	94 ± 13.1	92.5 ± 12.9	0.55	0.70

**Figure 2:**
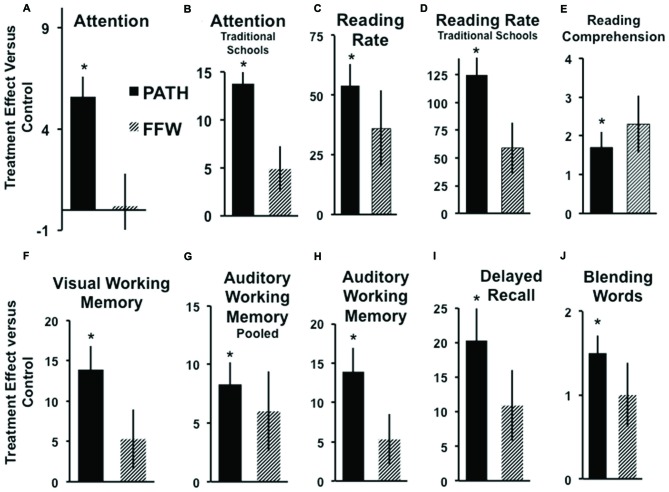
Improvements over controls in *Attention*: (A, pooled data), *p < 0.009, (B) Traditional Schools (TS), *p < 0.004; *Reading Speed*: (C, pooled data), *p < 0.004, (D) TS, *p < 0.007; *Reading Comprehension* Gray Oral Reading Test (GORT-3): (E, pooled data), *p < 0.046; *Visual Working Memory*: (F) TS, *p < 0.036; *Auditory Working Memory*: (G, pooled data), *p < 0.037, (H) TS, *p < 0.027; *Delayed Recall*: (I) TS, *p < 0.026; and *Phonological Processing* (Blending Words): (J, pooled data), *p < 0.0009, following each intervention: [*PATH to Reading* (*PATH)*: black, *FastForWord* (*FFW*): striped]. These barplots display the mean and (SE) difference in improvement of standardized scores in each treatment group compared to improvements observed in the control group. Positive bars indicate subjects in the treatment group improved more than subjects in the control group, negative bars mean control subjects improved more than those in the treatment group.

*Baseline values in Table 1 are mean ± SD for age and scores on tests graphed in Figure 2*.

So that the training could be done by one Research Assistant (RA) for each 1–2 second graders, 40 UCSD undergraduate RAs were trained extensively at the beginning of the school year. The RAs were in charge of administering the standardized tests and reading interventions. The standardized tests were administered in a masked manner; RAs did not know whether the student was in a control or treatment group, thereby removing the possibility of experimenter effects. Moreover, parents: (1) were not aware of what group their child was in; and (2) thought that the auditory or linguistic interventions would work more effectively, since these were the traditional interventions advertised to improve reading. This fact, though anecdotal, would suggest that if any parent expectancy effects were in play, they would work against the efficacy of the visual direction-discrimination intervention. Stickers were given to students at the end of training each day for good behavior and completing the intervention correctly to reward them for paying attention to the task. Motivational strategies were used to keep the participants on task.

### Experimental Design

The study was conducted for 20 weeks in four traditional schedule and 2-year-round public elementary schools in SDUSD in the morning, right before guided reading in the classroom, so that each student had plenty of practice on reading following the interventions. Twenty weeks of training was longer than used in previous studies of direction-discrimination training to improve reading fluency (Lawton, 2000, 2007, 2008, 2011), but was the minimum needed for the auditory timing training to be effective (Ostarello and De Ley, 2009). Controls were students who stayed in the classroom doing linguistic word building, when students in the treatment groups were pulled to do the visual or auditory timing interventions for 30 min, either 3 days a week for *visual timing* training or 5 days a week for *auditory timing* training. The linguistic word building intervention was a reading intervention provided by SDUSD, one aimed at improving phonics, decoding, vocabulary, reading comprehension, and reading fluency by word building exercises. Visual direction-discrimination was trained for a total of 20–30 h, depending on the time needed to complete 20 contrast thresholds, compared to 50 h of training on auditory phonemic processing. Auditory timing training was only done in schools having a traditional schedule, requested by those implementing the *auditory timing* training, since this training was found to regress in effectiveness when 4-week long vacations occur during the intervention training (Ostarello and De Ley, 2009), as occurs in the year-round schools.

At each school, the interventions were administered in a room devoted to this task on 13″ Macbook Pro computers purchased for this study. The computers were calibrated at the beginning of the school year with a Spectra Pritchard 1980A photometer to increase luminance and contrast linearly. The mean luminance was set to 125 cd/m^2^ on all computers by reducing the brightness of the screen 2–3 levels. The screen brightness, volume control, and date were checked each day before beginning visual timing training. Students sat an arm’s length from the screen, about 57 cm.

### Standardized Tests

Standardized tests of reading fluency, phonological processing, attention, and working memory were administered one-on-one to every student in the study before and after the intervention training by trained UCSD undergraduate students. These tests were chosen as the “Gold Standard” for fast and accurate measurements of fluency-based reading, attention, and working memory skills. The standardized tests which took about 1.5 h to administer were:

1. DESD using single word reading to measure the reading grade level, and spelling eidetically and phonologically to determine the level of dyseidetic (spelling) and dysphonetic (phononetic writing) deficits took about 10 min, distributed by Western Psychological Services in Los Angeles, CA, USA;
2. A computer-based reading speed assessment, described previously (Lawton, 2007) where six words of white text on a black background were presented each time from subsequent portions of an interesting story at increasing speeds, took about 3–5 min. The words were composed of large *sans-serif* letters. Text at their reading grade level, stories from Dr. Seuss, was used to measure reading speeds. Reading speed, measured in words/minute using a double staircase procedure, was not limited by the child’s rate of speaking, as is the case for the GORT below. In addition, words/minute is a much higher resolution scale, than the 1–5 scale used to score reading rates on the GORT-3 below;
3. (GORT-3) to measure both reading comprehension and reading speed took about 20 min;
4. Comprehensive Test of Phonological Processing (CTOPP) Blending Words subtest, using CD-based delivery, to measure phonological awareness, took about 5 min;
5. Cognitive Assessment Systems (CAS) test of Attention, measured using the Stroop and Number Detection subtests, took about 10 min;
6. Test of Information Processing Skills (TIPS) to measure both sequential (remembering the letters in the correct sequence) and nonsequential (remembering the letters in any order) auditory and visual working memory, and Delayed Recall took about 15 min.

### Interventions

#### Auditory Timing Intervention: FastForWord

The auditory timing intervention, implemented using FastForWord (FFW), developed by Scientific Learning Corporation, is designed to improve phonological processing by lengthening the phonemes until they are perceived accurately. The auditory timing intervention focuses on the building of auditory processing and oral language skills important for reading, by using acoustically-modified, digitally-generated speech: (1) frequency modulated tonal sweeps; (2) speech syllables with parametric modifications of temporal features, so format transitions were lengthened and intensified, or silent gaps were lengthened; (3) word exercises using human speech that was either parametrically modified in the temporal domain to teach students word ID, word matching, or following directions; and (4) phrases and sentences with increasingly complex grammatical structures to develop higher-level language skills, including phonology, morphology, syntax, grammar, and short-term memory. The stimuli changed adaptively, increasingly approximating normal speech, until the final levels, when normal speech was presented. This computer-based intervention is designed to build language and reading skills while strengthening the cognitive skills of memory, attention, processing, and sequencing. This auditory timing intervention was composed of seven exercises, three being done during each half-hour training session. To keep each participant challenged, but not frustrated, the exercises adapt so that the participant is successful around 80% of the time. Detailed reporting is provided to help instructors track participant performance, with alerts that let the instructor know when a participant is ready to move to the next product or when a participant is struggling and needs extra help. Individual interventions were provided for subjects who had difficulty progressing through the levels of the exercises to enable them to complete the exercises. For some subjects, occasionally the screen had to be covered, since the graphics prevented the subject from concentrating on the subtle auditory discriminations required to do* FFW* training. *FFW* was trained for 30 min, 5 days a week for 20 weeks.

#### Visual Timing Intervention: Motion Direction-Discrimination Training

The visual timing intervention, implemented using *PATH to Reading*, developed to remediate dorsal stream function, consisted of motion direction-discrimination training. This novel intervention (Lawton, 2000, 2007, 2008, 2011, 2015), developed by the author, and available commercially^1^, will be described in sufficient detail to understand its basic components. The student sat in front of a computer monitor with a display similar to the ones in Figure 1. During the presentation, the bars in the “fish-shaped” window in the center of the screen formed by a sinusoidal grating, moved left or right very briefly (450 ms). The student reported which way the center pattern moved by pushing the left or right arrow key. A brief tone was presented after incorrect responses. The program adaptively changed the contrast of the test pattern in order to keep the student at 79% correct. There were also levels of difficulty introduced by making the background pattern more similar to that in the fish, by increasing the pattern’s complexity level, and by increasing the number of directions of movement from one to two directions of motion.

The patterns used for this visual direction-discrimination task were designed to be optimal for activating magnocellular neurons (moving test pattern) relative to parvocellular neurons (stationary background; Lawton, 2000, 2007, 2011). In a given staircase run, the center spatial frequency (i.e., the test frequency) was either 0.25, 0.5, 1, or 2 cyc/deg. The surround grating spatial frequency was either equal to the test frequency or 1–2 octaves higher or lower than the test frequency, e.g., see Figure 1B. In addition to these simple backgrounds, multifrequency backgrounds were used, where the first background frequency equaled the spatial frequency of the single frequency background, having two additional background frequencies with a difference frequency equal to the test frequency. For example, the multifrequency backgrounds in Figure 1C are for a test frequency of 1 cyc/deg and a background frequency of 1 cyc/deg + 2 cyc/deg + 3 cyc/deg.

At the start of a session, both the test and background gratings were set to 5% contrast to ensure that the contrast of the test pattern was in the middle of the magnocellular contrast range (Kaplan and Shapley, 1986). Each time the child correctly identified the direction the fish stripes moved, the contrast of the test grating was lowered until the child answered incorrectly. Following the first incorrect response, a double-staircase procedure was used to estimate the direction-discrimination contrast threshold, which allowed measuring the contrast sensitivity, defined as the reciprocal of the contrast threshold times 100. This staircase procedure estimates the contrast needed for 79% correct responses, providing the most sensitive, repeatable measurements of contrast sensitivity (Higgins et al., 1984). A full training cycle of the direction-discrimination task required 20 threshold determinations (i.e., one for each of the four test spatial frequencies paired with each of the five background spatial frequencies).

The complexity level increased the: (1) number of sinewave components in the background from one (Figure 1B) to three (Figure 1C); (2) background contrast from 5% to 20%, (Figure 1C); and (3) pattern’s speed of movement after every four complexity levels, increasing from 6.7 Hz up to 13.3 Hz, as shown in Table 2, so that the student was challenged as the training progressed. The background contrast was increased to 20% contrast to provide a background that increased parvocellular activity, since magnocellular neurons saturate at 10% contrast (Kaplan and Shapley, 1986). The 20% contrast background required students to analyze information from magnocellular activity relative to increased parvocellular activity, making the task more challenging. The order of presentation for each complexity level was chosen to gradually increase the difficulty of the task (Lawton, 2011). Therefore, as the level of complexity increased, the contrast threshold should be higher initially. Once all 16 complexity levels of the *Motion* program were completed, the student progressed onto the next program, the *MotionMemory* program. Instead of discriminating the direction one pattern moved by pushing the left or right arrow key as in the *Motion* program, *MotionMemory* requires signaling the direction that two separate patterns moved, one after the other, by pushing one of four arrow keys. Each threshold in both the *Motion* and *MotionMemory* programs required 20–40 trials to complete. A score was given to make the training more game-like. The lower the contrast threshold, the higher was the score. After learning how to do this task, children typically took about 15–20 min to complete one replication, consisting of 20 contrast thresholds. Motion direction-discrimination was trained for between 15–30 min, 3 days a week for 20 weeks.

**Table 2:** **Stimulus characteristics at each complexity level**.

Complexity level	Pattern speed (Hz)	Background frequencies	Background contrast (%)
1	6.7	Single frequency	5
2	6.7	Multi frequency	5
3	6.7	Multi frequency	10
4	6.7	Multi frequency	20
5	8	Single frequency	5
6	8	Multi frequency	5
7	8	Multi frequency	10
8	8	Multi frequency	20
9	10	Single frequency	5
10	10	Multi frequency	5
11	10	Multi frequency	10
12	10	Multi frequency	20
13	13.3	Single frequency	5
14	13.3	Multi frequency	5
15	13.3	Multi frequency	10
16	13.3	Multi frequency	20

#### Linguistic Word Building Intervention (Control Intervention)

A linguistic word building intervention was implemented using *Learning Upgrade* to help struggling readers at the second and third grade level overcome reading difficulties. Each course contained 60 lessons sequenced to build reading skills. The lessons featured a song-video for instruction and a game for practice with remediation. After logging onto the website, a song-video of length 1–2 min was presented which taught the word building topic through lyrics with a catchy melody and a synchronized animated visual of letters, words and pictures. A game followed the song-video, which required students to answer a series of questions through interaction. Immediate remediation through a spoken voice and animated visuals was given for each incorrect answer, followed by additional problems. When a student reached 100 points, if they had achieved higher than 75% correct, they moved on to the next lesson. If not, they repeated the lesson with the same song but varied questions in the game. When a student completed all 60 lessons, they earned a Bronze certificate which could be printed. A student then used a visual map of lessons and scores to repeat any lessons below 90% to earn a Silver certificate, and then repeated any lessons below 95% to earn a Gold certificate. When a student had earned a Gold certificate, typically in about 20–30 h of time on task, the student was finished with the course and moved to a higher course.

### Hypotheses

The primary hypothesis in this study is that timing interventions: either one to improve auditory timing or one to improve visual timing, would improve attention, reading, and working memory more than linguistic word building exercises. Attention is the primary outcome measure, with reading speed and comprehension, phonological processing, and working memory being secondary outcome measures that result from improved selective and sustained attention. The secondary hypotheses, based on physiological data demonstrating that 1 cyc/deg is the lowest spatial frequency channel (Blakemore and Campbell, 1969), predict that direction discrimination sensitivity improves: (1) the most for the lowest spatial frequency channel, 1 cyc/deg, which moves twice as far in the same amount of time as the higher 2 cyc/deg test pattern; (2) the least for the 0.25 cyc/deg test pattern which requires pooling across spatial frequency channels to complete the task; and (3) more when a wider background frame of reference consisting of multiple spatial frequencies that are a harmonic (multiple) of the test frequency is presented, as found in typically developing observers (Lawton, 1989).

### Statistical Analyses

Change in test performance for the primary and secondary outcome measures (attention, reading speed and comprehension, working memory and phonological processing) and all secondary hypotheses were modeled using ANCOVAs controlling for age, sex, ethnicity (Caucasian, Hispanic, Asian, African-American), and school enrolled. Data was either: (1) pooled across schools having a traditional year schedule (four schools); or (2) pooled across the six schools, four having a traditional year schedule and two having a year-round schedule, with school and type of school (traditional vs. year round) included as covariates in the planned ANCOVA. ANCOVA contrast tests were used to compare change in standardized scores in controls vs. treatment groups.

A one-sample *t*-test was used to compare initial contrast sensitivity in the 58 dyslexics in this study to published levels in typically-developing second graders; paired *t*-tests were used to compare initial to final contrast sensitivity levels within each treatment group. The relationship between contrast sensitivity and the motion direction-discrimination task complexity level was assessed using 24 visual timing group students who completed all 16 levels of the* Motion* direction-discrimination training. For data from test frequencies 0.25, 0.5, 1.0, and 2.0 cyc/deg, the relationship between complexity level and contrast sensitivity was assessed using a linear mixed effects model, with hypothesis testing based on the fixed effect estimate of mean trajectory as complexity level increases, i.e., as amount of training increased. Tests investigating whether student’s contrast sensitivity at specific complexity levels deviated from the overall linear trend (i.e., were higher or lower than expected) were performed by adding indicator variables for the complexity levels in question to the linear mixed effects models. Paired *t*-tests were used to test for significant improvement in contrast sensitivity at 0.25, 0.5, 1.0, and 2.0 cyc/deg from baseline to end of study within students trained on *PATH to Reading*. All analyses were performed using the R statistical programming language. ANCOVA models were fit using the aov function (Chambers et al., 1992), and mixed effects models were fit using the lmer function (Pinheiro and Bates, 2000). All tests were 2-sided since students could increase or decrease in academic skills, with significance level *α* = 0.05 for all testing.

## Results

This study, examining the efficacy of visual timing vs. auditory timing vs. linguistic word building training, found significant improvements in student’s attention, reading fluency, and working memory *only* following visual motion-discrimination training when compared to linguistic word building. If language-based deficits underlie dyslexia, then training to improve auditory timing should also significantly improve these academic skills, since this training was done using clever, engaging auditory exercises for twice as long, 30 min 5 times/week, compared to the training to improve visual timing, done for 15–30 min 3 times/week.

### Effect of Interventions on Attention, Reading Fluency, and Working Memory

Students trained on motion direction-discrimination improved significantly more than controls, see Table 3 and Figure 2, in *Attention*: Figure 2A (pooled data) [*t*_(44)_ = 2.69, *p* = 0.009], and Figure 2B TS [*t*_(21)_ = 3.18, *p* = 0.004], *Reading Speed*: Figure 2C (pooled data) [*t*_(44)_ = 3.01, *p* = 0.004], and Figure 2D TS [*t*_(21)_ = 2.98, *p* = 0.007], *Reading Comprehension*: Figure 2E (pooled data) [*t*_(44)_ = 2.04, *p* = 0.046], sequential *Visual Working Memory*: Figure 2F TS [*t*_(21)_ = 2.34, *p* = 0.036], nonsequential *Auditory Working Memory*: Figure 2G (pooled data) [*t*_(44)_ = 2.14, *p* = 0.037], and Figure 2H TS [*t*_(21)_ = 2.34, *p* = 0.027], *Delayed Recall*: Figure 2I TS [*t*_(21)_ = 2.39, *p* = 0.026], and* Phonological Processing* (CTOPP Blending Words): Figure 2J (pooled data) [*t*_(44)_ = 3.52, *p* = 0.0009], whereas students trained on improving auditory timing, implemented using *FFW*, did not improve significantly more than controls on these tasks. The significant improvements in attention by students trained on visual direction-discrimination which required less attention to complete than either the auditory or linguistic intervention, shows that visual training is more effective than auditory or linguistic training in improving the attention networks. Visual training that does not activate dorsal stream functioning at both low and high levels, e.g., motion coherence, however, is not effective in improving reading fluency (Solan et al., 2004).

**Table 3:** **Mean increase in timing interventions (treatment effect) vs. word building intervention (controls) and the df (degrees of freedom), *t* value, and *p* value for the significant improvements only found following PATH training**.

Treatment effect vs. controls	PATH	FFW	df	*t* value	*p* value
Attention—pooled data	5.7 ± 1	0.2 ± 1.6	44	2.69	0.0009
Attention—traditional schools (TS)	13.8 ± 2.2	4.9 ± 2.4	21	3.18	0.004
Reading speed (wpm)—pooled	54 ± 9	36 ± 16	44	3.01	0.004
Reading speed (wpm)—TS	125 ± 21	59 ± 23	21	2.98	0.007
Reading comprehension—pooled	1.5 ± 0.4	2.3 ± 0.8	44	2.04	0.046
Sequential visual memory—TS	13.9 ± 2.9	5.3 ± 10	21	2.34	0.036
NonSequential auditory memory—pooled	8.3 ± 1.9	6 ± 3.4	44	2.14	0.037
NonSequential auditory memory—TS	13.9 ± 3	5.3 ± 3.3	21	2.34	0.027
Delayed recall—TS	20.3 ± 4.7	10.9 ± 5.1	21	2.39	0.026
Phonological awareness—pooled	1.5 ± 0.2	1.0 ± 0.4	44	3.52	0.0009

Direction-discrimination training improved reading speed in the classroom from 50 to 125 words/minute on average more than found using linguistic interventions. Note that even though both the auditory and visual timing groups had only six subjects in each group in TS, the visual timing intervention improved attention, reading speed, visual and auditory working memory, and delayed recall more than found when trained on the auditory timing intervention, and significantly more than found when trained using linguistic word building, implemented using *L*ea*rning Upgrade*. This same pattern of results was found the following year, yet they were not as large in magnitude, and only reading speed, phonological processing (Blending Words subtest of the CTOPP and Auditory Working-Memory) improved significantly, since the PATH intervention had to be administered before school, instead of before guided reading. *PATH to Reading* is most effective when immediately followed by guided reading. Moreover, even though motion direction-discrimination is a visual task, it significantly improved phonological processing more than interventions using an auditory task (either auditory timing or linguistic word building). Only students trained on motion direction-discrimination improved significantly more than controls in the combined (sequential and nonsequential) auditory working memory standardized score [*t*_(44)_ = 2.23, *p* = 0.03]. Future studies with larger sample sizes are needed to determine the advantage of improving visual timing over auditory timing conclusively.

### Effect of Interventions on Visual Motion Processing

**Figure 3:**
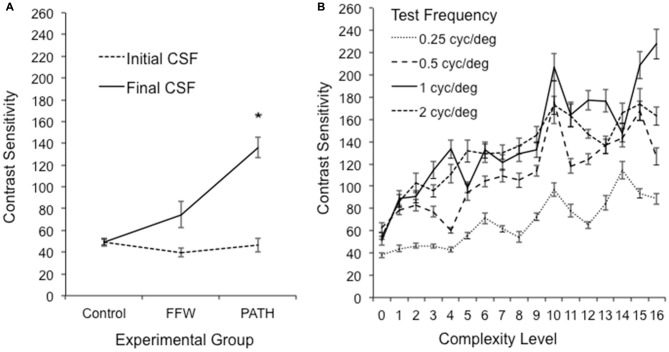
**(A)** Mean and (SE) improvements in *direction-discrimination* contrast sensitivity for 1 cyc/deg test patterns at first complexity level, plotting initial contrast sensitivity function (CSF) measured at the beginning and end of intervention training, averaged over the five different background patterns, for students in the *PATH* group, *significant at *p* < 0.0001. **(B)** Pooled data from traditional and year-round schools (26 subjects). Improvements in *direction-discrimination* contrast sensitivity at increasing levels of complexity, plotting initial (0) and maximum contrast sensitivity at each level of complexity (1–16) for each of the four test frequencies: 0.25, 0.5, 1 and 2 cyc/deg. The data in this graph represent the mean and (SE) contrast sensitivity averaged across subjects trained on *PATH* in TS (6) and year-round schools (20) for subjects who completed all 16 levels of complexity in the *PATH* program.

Students in this study had abnormal visual motion processing, as shown by the mean baseline Contrast Sensitivity Function (CSF) for direction discrimination in Figure 3A. Initially participants in this study had elevated contrast thresholds for movement discrimination, averaging 2.9% ± 0.2, significantly higher [one sample *t*_(44)_ = 5.81, *p* < 0.0001] than the previously reported mean contrast threshold for typically-developing second graders of 1.35% ± 0.1 (Lawton, 2007). Direction-discrimination contrast sensitivity improved significantly only for those students who were trained on the motion direction-discrimination intervention (Figure 3A), improving in sensitivity three-fold after motion direction-discrimination training in both TS [one-sample paired *t*_(5)_ = 3.694, *p* = 0.014] and in pooled data from traditional and year-round schools, [one-sample paired *t*_(24)_ = 5.618, *p* < 0.0001]. The motion discrimination CSF increased significantly as a function of complexity level for each of the test frequency targets, shown in Figure 3B and Table 4. The temporal frequencies that the students could not discriminate the direction of movement before training, and had the highest contrast sensitivities following training were the 10 and 13 Hz motion (complexity levels 9–16).

**Table 4:** **Mean increase in contrast sensitivity as a function of test frequency and PATH complexity level for pooled data from traditional and year-round schools**.

Test frequency	Mean increase per complexity level	Standard error	*t*_(22)_ value	*p* value
0.25 cyc/deg	4.19%	0.59%	7.11	<0.0001
0.5 cyc/deg	7.29%	0.85%	8.56	<0.0001
1 cyc/deg	11.22%	1.54%	7.26	<0.0001
2 cyc/deg	7.83%	1.35%	5.79	<0.0001

When the 5% contrast background changed from being single frequency to being composed of multiple spatial frequencies (i.e., at complexity levels 2, 6, 10, and 14, described in Table 2), contrast sensitivity levels were higher, on average, 18.5 points relative to the general linear trend [*t*_(21)_ = 2.62, *p* = 0.016]. Conversely, when the multifrequency background pattern was presented at 20% contrast for the lowest spatial frequency target of 0.25 cyc/deg (complexity levels 4, 8, 12, and 16 in Table 2), contrast sensitivity was lower, being marginally significant [*t*_(21)_ = −1.99, *p* = 0.06], which is expected because at 20% contrast, the background activates parvocellular neurons more than magnocellular neurons (Kaplan and Shapley, 1986), making the task more difficult. The 0.25 cyc/deg test frequency requires pooling of contrast information over several spatial frequency channels since, as shown by Blakemore and Campbell (1969), there are no luminance-varying spatial frequency channels below 1 cyc/deg. Since the 1 cyc/deg test pattern showed the highest mean increase in contrast sensitivity, 11.2% per level of complexity, this indicates that the 1 cyc/deg test pattern, is the predominant test frequency for improving motion discrimination. Finding an increased CSF at increasing levels of complexity, thereby increasing: (1) the speed of motion, as shown in Table 2; and (2) the width of the background frame of reference (from single to multifrequency backgrounds) and its contrast (activating more parvocellular neurons at higher contrasts) suggests that direction-discrimination training improves the functioning of magnocellular neurons (left-right movement) relative to the functioning of parvocellular neurons (stationary background).

Not only was contrast sensitivity for direction-discrimination increased significantly following motion training, but also the time to discriminate the direction of motion was reduced significantly for students who did the direction-discrimination intervention. For example, for the 1 cyc/deg test frequency, the most sensitive test frequency target (see Figure 3B), the mean time to complete five threshold measurements decreased an average of 6 s per complexity level [mean decrease by mixed effects model analysis *t*_(22)_ = 4.225, *p* = 0.004]; mean time was 5.00 min ± 0.22 at baseline and 2.38 min ± 0.17 at complexity level 16. That is, the mean time to complete motion direction-discrimination decreased as the complexity level increased. These results show that both the: (1) sensitivity to discriminate direction-discrimination increased; and (2) time required to complete motion direction-discrimination training decreased.

## Discussion

The key stimulus attribute needed to detect motion discrimination deficits in dyslexics is assessed by measuring the contrast sensitivity for the direction of motion relative to a stationary background (Georgeson and Scott-Samuel, 1999). Only when the direction of motion is discriminated against a stationary background do both dysphonetic and dyseidetic dyslexics exhibit an impaired ability to discriminate the direction of motion (Lawton, 2000, 2007, 2011; Ridder et al., 2001). When the direction of movement is not judged relative to a stationary background, then some dyslexics do not exhibit motion deficits, as reviewed previously (Stein, 2001; Gori et al., 2014). Studies that have questioned whether magnocellular deficits in the dorsal stream cause the reading problems found in dyslexics (Amitay et al., 2002; Williams et al., 2003) examined a dyslexic’s sensitivity to flicker or high contrast random dot patterns, relative to *no* background pattern or a moving background (Sperling et al., 2006), none of these stimuli being optimal for activating direction-selective cells (Baker, 1990; De Valois et al., 2000). Patterned backgrounds, as opposed to featureless backgrounds, require figure/ground discrimination, suggesting that a core deficit in dyslexia may be figure/ground discrimination analyzed by the dorsal stream, consistent with: (1) the dyslexic’s deficits being primarily due to deficits in the spatiotemporal parsing of the letter stream (Vidyasagar, 1999, 2001) that are normally transmitted both by feedforward magnocellular (low-contrast movement) input and feedback at the attended location from LIP to MT (Saalman et al., 2007) and from MT to V1 (Hupe et al., 1998); (2) an impairment in the low gamma frequency oscillations reducing feedback in visual cortical areas (Vidyasagar, 2013); and (3) in excluding noisy backgrounds (Sperling et al., 2006). Training with the stationary background frame of reference provided by multifrequency backgrounds (Figure 1C) improved the dyslexic’s ability to discriminate the direction of movement (Lawton, 2011), enabling the child to attend to a wider region of space. This study supports the hypothesis (Lawton, 1989) that stationary multifrequency backgrounds confer an advantage when discriminating the direction of motion, providing a wider, more structured frame of reference, most likely by taking advantage of MT’s center-surround organization (Allman et al., 1985) to facilitate figure/ground discrimination. Moreover, only with stationary textured backgrounds has motion direction-discrimination training been found to improve reading fluency in all types of dyslexics (Lawton, 2000, 2007, 2011).

This study found that direction-discrimination training, a task that optimally activates the V1-MT network (De Valois et al., 2000), improved: (1) movement direction sensitivity; (2) speed of processing for both motion direction discrimination and reading rates; (3) attention; (4) reading comprehension; (5) phonological processing; and (6) both auditory and visual working-memory, including delayed recall, more than found following phonological training, either by improving auditory timing or word building strategies. These results indicate that direction-discrimination training improves the sensitivity and timing of sluggish magnocellular neurons (improving dorsal stream function), relative to parvocellular neurons early in the dorsal stream, as evidenced by improved motion discrimination sensitivity at higher background contrasts and temporal frequencies. After direction-discrimination training, the highest contrast sensitivities were found for patterns moving from 10–13 Hz, these temporal frequencies being key to improving attention in dyslexics. These results contradict Goswami’s temporal sampling framework theory, proposing that the key timing deficits in dyslexia are for movement <10 Hz (Goswami, 2011). This study found that improving visual motion direction-discrimination sensitivity and timing improved processing in the neural networks underlying attention, reading, and working memory in dyslexics. These improvements are found by presumably improving low levels in the dorsal stream, the V1-MT network, which improved functioning at higher levels in the dorsal stream, including the PPC, the dorsal lateral prefrontal cortex (DLPFC), and the attention networks. This study provides additional evidence that *visual* motion processing is fundamental for paying attention, good reading performance, and remediating reading deficits, contrary to common practice based on the assumption that only auditory-based phonological processing can be used to remediate reading deficits (Tallal et al., 1993; Temple et al., 2003; Vellutino et al., 2004; Dehaene, 2009; Olulade et al., 2013).

Initially, the biological basis of dyslexia was assumed to be in the brain regions responsible for the visual perception of text (Hinshelwood, 1917). However presently, the dominant view is that the core deficit underlying reading disabilities is an auditory phonological processing deficit (Bradley and Bryant, 1983; Tallal et al., 1993; Temple et al., 2003; Vellutino et al., 2004; Dehaene, 2009; Olulade et al., 2013). A careful examination of the neuroimaging studies responsible for this paradigm shift reveal that visual word form areas and other visual processing areas were also implicated in many of these studies. For instance, Shaywitz et al. (1998) state “Brain activation patterns differed significantly between the groups with dyslexic readers showing relative underactivation in posterior regions (Wernicke’s area, the angular gyrus, and striate cortex) and relative overactivation in an anterior region (inferior frontal gyrus).” Finding the striate (visual) processing area to be hypoactive in persons with dyslexia is widespread in the literature (Eden et al., 1996; Demb et al., 1998; Shaywitz et al., 1998; Shelley-Tremblay et al., 2011) and reliably co-occurs with abnormal patterns of cortical activity in areas more typically associated with auditory analyses. The visual contribution of dorsal stream processing to dyslexia has been dismissed by the American Academy of Pediatrics (2009) based on a version of the magnocellular deficit theory that has been shown to be biologically implausible (Scarborough, 2005). Proposing that phonological processing deficits are the sole and key abnormal factor in dyslexia is not born out by studies showing visual motion processing deficits are found for all types of dyslexics (Lawton, 2000, 2007, 2011; Ridder et al., 2001). While phonological processing is a reliable and robust predictor of future reading, it cannot fully account for the variance in reading ability and the full range of deficits in dyslexic readers, instead only accounting for approximately 25% of future reading skills (Mann and Liberman, 1984; Wagner, 1997).

### Novel Method to Remediate Attention, Reading, and Working Memory in Dyslexics

Movement figure/ground discrimination, a novel method (Lawton, 2000, 2015), is fundamental for detecting and remediating attention, reading, and memory problems for *all* types of dyslexics. This study found that training to improve motion direction-discrimination, most likely by improving the timing and sensitivity of directionally-selective magnocellular neurons relative to parvocellular neurons in the dorsal stream is linked to improved attention skills, enabling the beginning and end of the word, and processing the letters sequentially to be done effortlessly, thereby improving reading performance. Moreover, previous studies (Lawton, 2011) found that the more a student was trained on motion direction-discrimination, the more reading speed improved. Consequently, abnormal visual motion processing is implicated as a fundamental factor underlying the reduced functionality of the attention networks in dyslexics, causing slow reading speeds and poor comprehension. Furthermore, this abnormality can be remediated rapidly by visual training that improves a person’s contrast sensitivity for direction-discrimination of dim vertical bars moving relative to a stationary textured background, indicating that visual timing deficits are a cause not a result of dyslexia.

The significant improvements in both phonological processing and auditory working-memory found in this study demonstrate that training to improve visual timing improves auditory skills. Consequently, training early in the visual dorsal stream improved higher levels of processing in the dorsal stream, in particular the PPC, where: (1) there is a supramodal representation of space with convergence of both auditory and visual inputs in the parietal cortex (Farah et al., 1989); and (2) selective endogenous attention activates this area which connects to frontal areas, like the DLPFC (Posner et al., 1984; Posner and Petersen, 1990; Supekar and Menon, 2012). By improving attention, students were able to hear the sequential ordering of sounds more accurately, improving phonological processing and auditory working memory. Students given training aimed at auditory magnocellular function, as embodied by the auditory timing intervention, improved in reading fluency, but the improvements were not significant when compared to the improvements made by controls, as also found in a review of *FFW* studies (Strong et al., 2011).

A major limitation of this study is the small sample size in the group to improve auditory timing. Most of the students in our study were from year-round schools, whose schedules precluded implementing the auditory timing program. Power to detect treatment effects in the auditory timing group was limited, requiring a larger study to determine unequivocally the relative effect of improving auditory timing on reading fluency and attention. Another limitation of this study is the lack of an out of classroom control condition that was comparable in terms of the extent of personal attention from the college students administering the reading interventions. Since half the classroom was pulled to be trained on the timing interventions, the students who stayed in the classroom had much more attention from their classroom teacher. Moreover, there was no evidence of such an effect in the auditory timing group, even though this group experienced the same level of personal interaction with students for 5 days a week compared to only 3 days a week for the visual timing intervention. Hence, it is unlikely that the significant effects observed in the motion direction-discrimination group were driven by effects associated with pulling children from the classroom and personal attention.

This study found that motion direction-discrimination training remediates reading deficits of *both* phonological (requiring accurate temporal sequencing) and visual (requiring accurate spatial sequencing) origin. Moreover, there is evidence that improvements in reading speed after motion direction-discrimination training are sustained over time (Lawton, 2011), whereas improvements in word reading found following auditory interventions to improve phonological processing degrade over time, two years later showing no difference in word reading compared to controls not having the auditory intervention (Wise et al., 2000).

### Sluggish Magnocellular Processing Limits Reading Acquisition in Dyslexics

It has been proposed that the visual system exploits the dichotomy of a fast magnocellular channel and a slower parvocellular channel for the purpose of selective attention (Vidyasagar, 2001, 2012, 2013). The faster transmission time of the magnocellular neurons projecting predominantly to the dorsal stream are ideal to provide the input for feedback to intermediate stages in the cortical dorsal and ventral streams, as well as to V1 (Vidyasagar, 1999, 2001, 2013). Feedback from MT has its strongest effects for stimuli of low salience (Hupe et al., 1998), such as the low contrast patterns that maximally activate magnocellular neurons (Kaplan and Shapley, 1986; Sclar et al., 1990) that are being used to train visual movement discrimination in this study. There is parvocellular input to MT from: (1) parvocellular layers in the lateral geniculate nucleus (Nassi et al., 2006); (2) layer 6 V1 cells, having both parvocellular and magnocellular input, projecting to layer 4Cb in V1 which projects to MT (Callaway, 1998); and (3) V4 (Maunsell et al., 1990), enabling parvocellular activity to provide a background frame of reference for discriminating the direction of movement in the dorsal stream. Parvocellular functioning among dyslexics has been found to be equivalent to that in normal controls, whereas magnocellular function is significantly impaired (Lovegrove et al., 1980; Hansen et al., 2001; Kevan and Pammer, 2009; Gori et al., 2014), being the primary cause for slow reading and attention deficits.

**Figure 4:**
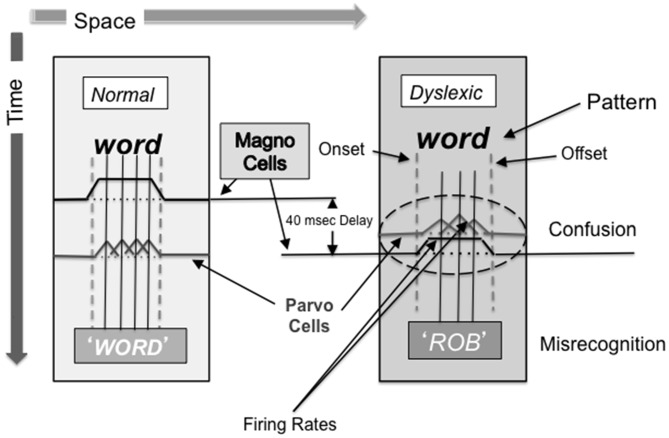
**Word distortions resulting from sluggish magnocellular neurons**. What happens when the pattern and motion pathways are not working together: dyslexics always go to the middle of word, often not seeing the first letter and flipping the order of the letters. The dorsal stream sets up frame of reference (where is word located) and ventral stream analyses details sequentially. The dyslexic appears to tunnel down to get meaning (individual letters), not knowing where to go next with their eyes since magnocellular neurons are sluggish, being delayed 20–40 ms, causing confusion and misrecognition.

When reading, it has been proposed that the PPC uses the spatial information of the location and overall shape and form of a word it receives through the rapid magnocellular pathway to gate the information going into the temporal stream. The information is gated via attentional feedback to the striate cortex and to other regions in the occipito-temporal cortex (Martinez et al., 1999; Vidyasagar, 1999, 2001), most likely by top-down feedback which uses synchronized neuronal oscillations at the lower end of the gamma frequency range (Vidyasagar, 2013), which can then be used by parvocellular neurons in the ventral stream as a starting point for deciphering the individual letters (Vidyasagar, 2001; see Figure 4). Each cycle of gamma oscillation focuses an attentional spotlight on the primary visual cortical representation of just one or two letters before sequential recognition of these letters and their concatenation into word strings. The timing, period, envelope, amplitude, and phase of the synchronized oscillations modulating the incoming signals in the striate cortex have a profound influence on the accuracy and speed of reading (Vidyasagar, 2013). The speed determined by the gamma frequency oscillation is the essential rate-limiting step in dyslexia (Vidyasagar, 2013). Figure/ground movement discrimination training is likely to strengthen coupled: (1) theta/gamma activity for the test patterns moving at 6.7 and 8 Hz; or (2) alpha/gamma activity for the test patterns moving at 10 and 13.3 Hz. Therefore, it is likely that the visual direction-discrimination training paradigm used in this study improves not only magnocellular function and attention, but also magno-parvo integration, figure/ground discrimination, and low gamma frequency oscillation.

Our working hypothesis is that sluggish magnocellular neurons early in the dorsal cortical visual pathway (V1), found in dyslexics (Livingstone et al., 1991), disrupt processing at higher levels of dorsal stream processing, dyslexics having little or no activity in MT (Eden et al., 1996; Demb et al., 1998), including the development of these processes. After 6 weeks of motion direction-discrimination training 3 times/week in dyslexic fourth graders, dorsal stream activity improved as shown by their Visual Evoked Potentials (Shelley-Tremblay et al., 2011), consistent with a recent pilot study using magnetoencephalography (MEG) source imaging (Lawton and Huang, 2015) that found improved function in both the dorsal stream (V1, V3, MT, MST areas) and fronto-parietal attention networks. Magnocellular output from the anterior portion of the dorsal stream, including the PPC, is input to the mid/posterior insula, a hub of the Central Executive Network (CEN), which includes the PPC and the DLPFC (Supekar and Menon, 2012). Magnocellular activity signals the beginning and end of a word, thereby gating the processing of parvocellular activity, as proposed by Vidyasagar (2001, 2013), and illustrated in Figure 4. The sluggish magnocellular neurons in dyslexics not only result in attention deficits, an impairment in the low gamma frequencies reducing feedback in visual cortical areas (Vidyasagar, 2013), but also disrupted processing in LIP and FEF, either within a fixation, between fixation sequences, or both (Vidyasagar, 2001; Slaghuis and Ryan, 2006; Fischer, 2012). This study found, for the first time, that direction-discrimination training improved not only reading fluency, but also attention and working memory. Therefore, direction-discrimination training improved CEN functioning, also found using MEG source imaging (Lawton and Huang, 2015), providing more evidence that abnormal visual motion processing is a fundamental cause of attention and subsequent reading problems in dyslexics.

By improving the attention network’s functioning, motion direction-discrimination training provides a wider usable field of view so that more objects are perceived in their correct location in a single glance. Motion direction-discrimination training is the key for reading acquisition to happen at an efficient speed for dyslexics, most likely by increasing the ease of magno-parvo integration. When motion direction-discrimination training was followed by guided reading in the classroom, attention, reading fluency, and working memory skills improved significantly more than found after training on linguistic word building or auditory timing interventions. Remediating visual timing deficits in the dorsal stream reveals the causal role of visual motion discrimination and attention in reading acquisition. This study supports the hypothesis that faulty timing in synchronizing the activity of magnocellular (left-right movement discrimination) with parvocellular (stationary background) visual pathways are a fundamental cause of dyslexia and argues against the assumption that reading deficiencies in dyslexia are caused by phonological or language deficits. This study demonstrates that visual movement figure/ground discrimination can be used to not only detect dyslexia early, but also for its successful treatment, so that reading problems do not prevent children from readily learning.

## Author Contributions

TL designed study, recruited and trained staff, ran daily operations, and wrote article.

## Conflict of Interest Statement

The author has a potential conflict of interest, since she is the developer of Path To Reading (PATH). She had no part in collecting or analyzing the data, thereby having no influence over the results we obtained.

## Footnotes

^1^www.pathtoreading.com
